# Nurse-administered intravitreal injections of anti-VEGF: study protocol for noninferiority randomized controlled trial of safety, cost and patient satisfaction

**DOI:** 10.1186/s12886-016-0348-4

**Published:** 2016-10-01

**Authors:** Dordi Austeng, Tora Sund Morken, Stine Bolme, Turid Follestad, Vidar Halsteinli

**Affiliations:** 1 Department of Ophthalmology, St. Olavs Hospital, Trondheim University Hospital, Trondheim, Norway; 2 Regional Center for Healthcare Improvement, St. Olavs Hospital, Trondheim University Hospital, Trondheim, Norway; 3 Department of Neuroscience, Norwegian University of Science and Technology (NTNU), Trondheim, Norway; 4 Department of Laboratory Medicine, Children’s and Women’s Health, Norwegian University of Science and Technology (NTNU), Trondheim, Norway; 5 Department of Public Health and General Practice, Norwegian University of Science and Technology (NTNU), Trondheim, Norway

**Keywords:** Anti-VEGF, Intravitreal injection, Nurse, Randomized controlled trial, Age-related macular degeneration, Retinal vein occlusion, Diabetic macular edema

## Abstract

**Background:**

Intravitreal injections (IVI) of anti-vascular endothelial growth factor (anti-VEGF) now improve or stabilize visual acuity in a number of previously untreatable eye diseases, of which the main are age-related macular degeneration, retinal vein occlusion and diabetic macular edema. Most patients require multiple injections over lengthy periods of time and the prevalence of treatable conditions is increasing. Anti-VEGF IVI normally administered by physicians, therefore represent a considerable workload on ophthalmologic clinics and will continue to do so in the near future. Nurse-administered IVI may relieve this workload, but the safety, cost and patient satisfaction of such an extended role for nurses in ophthalmologic clinics has not earlier been investigated. To investigate these outcomes following independent anti-VEGF IVI by trained nurses, a noninferiority randomized controlled trial is being conducted.

**Methods/Design:**

Patients eligible for anti-VEGF treatment, minimum 304, are recruited and randomized to IVI administration by either trained nurses or physicians. The primary outcome is safety, measured by difference in mean change in visual acuity between the two groups during an observation period of 12 months. Secondary outcomes are incidence of ocular adverse events, cost per patient and patient satisfaction.

**Discussion:**

This study protocol describes the design of the first randomized controlled trial of nurse-administered IVI of anti-VEGF. The study is designed to examine safety, cost and patient satisfaction during 12 months follow-up.

**Trial registration:**

ClinicalTrials.gov NCT02359149. Registered February 4, 2015.

**Electronic supplementary material:**

The online version of this article (doi:10.1186/s12886-016-0348-4) contains supplementary material, which is available to authorized users.

## Background

Intravitreal injections (IVI) of anti-vascular endothelial growth factor (anti-VEGF) improve or stabilize visual acuity in a number of previously untreatable eye diseases, of which the main are age-related macular degeneration (AMD), retinal vein occlusion (RVO) and diabetic macular edema (DME) [1–3]. Due to its potent antiangiogenic effects, the number of IVI of anti-VEGF has risen considerably since the treatment was first introduced a decade ago [4, 5]. Elderly people with AMD make up the largest group of patients receiving IVI and the prevalence of the disease increases with age. In the UK 3.5 % of the population of 75 years or older were visually impaired due to AMD [6]. In 2010, more than two million people were blind and six million people were visually impaired due to macular diseases globally [7]. Improved diagnostics as well as increased prevalence of treatable conditions will probably cause a continued rise of IVI in the future. IVI are normally administered by physicians in ophthalmologic out-patient clinics and are given with intervals of 4–16 weeks either as monthly injections, injections when needed (pro re nata) or injections with gradually extended intervals (treat and extend) [8, 9]. Irrespective of the treatment strategy chosen, most patients require sequential injections during several years for their condition to stabilize. Hence, IVI of anti-VEGF represent a considerable workload on physicians in ophthalmologic clinics and is expected to continue to do so. This is a real challenge today and in the future, since the population over age 60 are growing more than twice as fast as the number of ophthalmologists [10].

Extended roles for nurses are increasingly implemented in several medical fields, and in ophthalmology nurse-administered IVI of anti-VEGF may replace physician-administered IVI. Data on nurse-administered IVI is limited so far, but there are indications that it may be safe and acceptable to patients. An observational study from the UK reported a complication rate comparable to studies in which IVI were administered by physicians [11] and other studies have reported acceptable patient satisfaction following nurse-administered IVI [12–14]. However, these outcomes have to the best of our knowledge not earlier been investigated in randomized controlled trials (RCT) and an economic evaluation of a nurse-administered IVI clinic has not earlier been reported. To this end, the present protocol describes a noninferiority RCT with the objective to investigate safety, cost and patient satisfaction following nurse-administered IVI during 12-months follow-up.

## Methods

### Study design

**Fig. 1:**
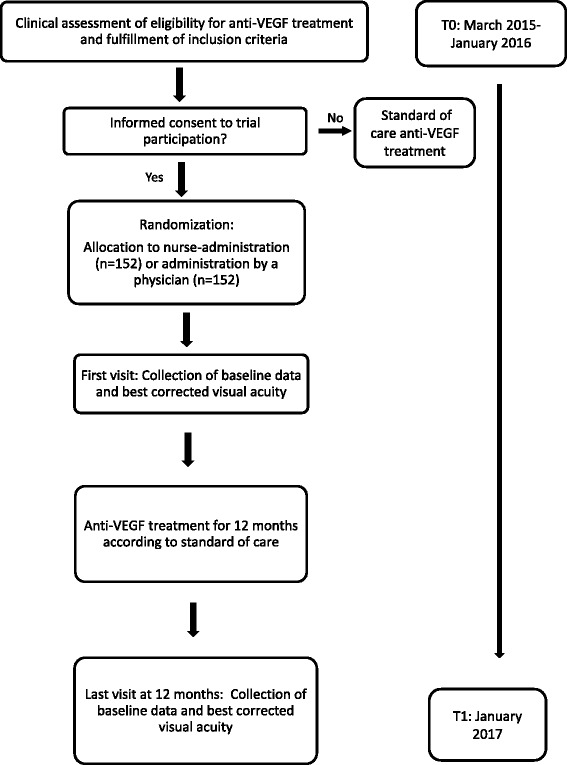
Flowchart. Overview of the enrollment and follow-up of study participants

The study is a prospective, randomized noninferiority trial with two treatment arms; IVI performed by nurses and IVI performed by physicians. Treatment by physicians is considered the reference group and standard care to which treatment performed by nurses will be compared. The flow chart of the study is presented in Fig. 1.

### Objectives

The primary objective is to evaluate the safety of nurse-administered IVI of anti-VEGF compared with physician-administered IVI.

The secondary objectives are:

- to evaluate cost of nurse-administered IVI of anti-VEGF compared with standard care
- to evaluate patient satisfaction of nurse-administered IVI of anti-VEGF compared with standard care

For the primary objective the evaluation will be performed using a noninferiority test, to test whether the nurses are treating the patients equally safe or better than the reference group, i.e physicians. More specifically, the null (H_0_) and alternative (H_A_) hypotheses are:

$$ {\mathrm{H}}_0:\ {\upmu}_{\mathrm{NURSES}}\hbox{--}\ {\upmu}_{\mathrm{PHYSICIANS}}\le -{\updelta}_{\mathrm{L}}\kern0.37em \mathrm{and}\;{\mathrm{H}}_{\mathrm{A}}:\ {\upmu}_{\mathrm{NURSES}}\hbox{--}\ {\upmu}_{\mathrm{PHYSICIANS}}> - {\updelta}_{\mathrm{L}}, $$

where μ _i_ is the mean change in visual acuity from first visit (baseline) to last visit 12 months later in group i, and δ_L_ is “the noninferiority margin”, which is the maximum clinically acceptable difference in change, for treatment by nurses to be considered noninferior to the reference treatment, δ_L_ >0

### Setting

The trial is performed in the IVI clinic of the Department of Ophthalmology, St. Olavs Hospital, Trondheim University Hospital in Norway during 01.03.15-31.12.16. The IVI clinic, organized as an independent out-patient clinic, performs ~ 3000 IVI annually and serves a population of ~ 300.000 individuals in the Central Norway Health Region.

Patients are remitted to the IVI clinic by ophthalmologists working at 10 different eye centers in the region and from ophthalmologists at the Department of Ophthalmology, responsible for the diagnostic and therapeutic decisions. At the IVI clinic a secretary administers patient appointments, receive user charges and answer phone calls. Preparing the patients for IVI is performed by a nurse (Additional file 1). In standard care, a physician is responsible for performing the IVI according to the list of patients, approximately 22 IVI daily (Additional file 2). A senior consultant is available at the clinic in case medical questions need to be discussed.

### Intervention

The intervention in the present study consists of replacing the physician administering the IVI, by a nurse (Additional file 2). Administration of IVI includes several skills and responsibility: assessment of whether there are any contraindications for treatment, performing the sterile IVI procedure, informing the patient, planning the next session and documentation in patient records. The preparation for IVI by a nurse (Additional file 1) and the possibility to seek medical advice from a senior consultant remains equal to standard care.

### Training program

A training program for administration of IVI for nurses was developed and implemented at the Department of Ophthalmology during the year prior to the start of the RCT. Six nurses took part in the training program which aimed at enabling nurses to perform IVI independently, as safe and within equal time frame as the physicians.

The program included two interactive courses regarding eye infections and documentation in patient records. Furthermore, it included a wetlab with individual training of a safe injection technique on porcine eyes; placing the injection 3.5 mm posterior to the limbus in any quadrant between the horizontal and vertical muscles. This was followed by graded exposure of the procedure in the injection room and finally performing IVI individually on patients under the supervision of a physician. The training program was divided into steps with increasing difficulty and the participating nurses decided themselves when they were ready to move to the next step. The nurses had to perform 100 independent IVI before final certification. The final achieved competence was evaluated by an unbiased senior consultant via observation of the nurse performing three independent IVI. If these were performed in a satisfactory manner, the nurse was certified to administer IVI individually.

### Trial recruitment

All patients with AMD, RVO or DME that are eligible for anti-VEGF IVI and able to give an informed consent are invited to participate. Both newly referred patients during 01.03.15–01.01.16 as well as patients that are already receiving anti-VEGF IVI fulfil the inclusion criteria.

Patients with ocular pathology eligible for anti-VEGF IVI other than the abovementioned conditions and inability to provide an informed consent do not fulfil the inclusion criteria and are excluded.

Information about the study will be given either face-to-face in the department or via a phone call and will also be handed out in writing. The patients will receive trial information at least 24 h before being asked to give an informed consent. If the patient agrees to enter the trial, written informed consent will be obtained. Consent forms will be stored in a locked safe to which only study management has access. Patients who do not consent to the trial will be treated according to standard care.

### Randomization and blinding

Patients are randomly assigned to receive treatment by nurse or physician in a 1:1 ratio, using a web-based algorithm. The randomization is stratified by diagnosis (AMD, RVO and DME) and by number of treatments (first treatment vs treated before). The reason for choice of stratification is that there is an expected difference in change in mean visual acuity during the observation period in these groups. Only one eye per patient is included in the study. If both eyes are eligible, the eye with the better visual acuity is included.

The study is single-blinded, i.e. patients are blinded to intervention group. Patients are not told to which group they have been randomized, and both nurses and physicians will wear white hospital clothes but no nametags telling their profession during the procedure. Furthermore, when the patient enters the injection room the personnel will present themselves by first name only.

### Outcomes

Primary outcome is the difference in mean change in visual acuity between the two groups during the study period of 12 months (measure 1).

Secondary outcomes are:

- incidence of ocular adverse events needing treatment (measure 2)
- cost per patient (measure 3)
- mean patient satisfaction score (measure 4)

### Measures

1. *Visual acuity* is measured with the Early Treatment Diabetic Retinopathy Study (ETDRS) chart using a standardized testing protocol at a starting test distance of 2 m [4, 5]. The visual acuity is measured as number of letters read at the ETDRS chart. Each line of the chart has five letters of same size in each row. The letters of the following rows gradually become smaller, with a distance of 0,1 logMAR. This interval scale is considered a continuous variable, and the number of letters read is counted [15]. The mean number of letters scored is considered a precise measure for evaluating whether the intervention shifts the visual acuity compared to standard care.

The test is carried out under uniform conditions by a physician, orthoptist or an optician. Before testing, the refraction is corrected following a standard protocol [15], i.e. the vision tested being the best corrected visual acuity.

2. *Ocular adverse events*. Number of ocular adverse events in the population receiving IVI at the Department of Ophthalmology is recorded during the whole study period, from the first study visit to the last follow-up visit of the study. The ocular adverse events will be noted in patient record and on a dedicated study form. Only ocular adverse events needing treatment are being recorded; retinal detachment, retinal tears, endophthalmitis, uveitis, lens damage and intraocular hemorrhages.
3. *Cost per patient*. Cost data will be collected in order to take a hospital perspective, a health care perspective and a societal perspective.

#### Intervention costs

The calculation of out-patient clinic costs will be based on time spent by different personnel categories. Time spent will be recorded according to the three main phases of the treatment procedure: Pre-examination, the IVI-procedure and the post IVI-procedure:

- Pre-examination services performed by secretaries and nurses
- IVI-procedure performed by nurse (intervention) or physician (standard care) and time spent by senior consultant on on-call assistance to the nurse or physician respectively.
- Post-IVI services performed by secretaries.

Number of hours spent will be multiplied by personnel group specific salary levels and adjusted with over-head costs. Data will be recorded on a daily basis using predefined registration forms (Additional file 3). Aggregate costs per patient will be calculated.

Extra educational costs on training nurses will be calculated based on the training program.

#### Other hospital costs

Utilization of hospital services outside the out-patient clinic will be assessed by examining data from the hospital administrative patient register. Costs will be calculated by combining volume of in-patient and out-patient services and their corresponding unit costs.

#### Health care costs outside hospital

Utilization of ophthalmologist services and general practitioner services will be collected using a patient questionnaire (Additional file 4). Costs will be calculated by combining volume of services and corresponding unit costs.

#### Patient costs

Travel costs will be calculated based on information on travel time and bringing a companion.

4. *Patient satisfaction*. Previously validated patient satisfaction instruments were found too comprehensive and not suitable to assess the IVI treatment in the injection room setting. A short and simple study-specific patient satisfaction questionnaire was therefore developed in accordance with guidelines for measuring the quality of health services [16]. The questionnaire was validated for reliability and feasibility in a pilot study of 10 patients. After this first pilot test, some modifications were made to the questionnaire before a second pilot test was carried out and validated. We found the best alternative for the patients with blurred vision following treatment to be a five-point grading scale and only a few questions read out load. Only two aspects of the treatment is tested; the general impression of the treatment and the confidence during the treatment in the injection room (Additional file 5). If the patient is not giving the maximum score of satisfaction, an open-ended question will be asked for recommendations of how to improve the comfort and well-being during the visit. At the last visit, the patients additionally will be asked if they think they are treated by a nurse or by a physician.

### Data collection

At the first visit, background information for the economic evaluations is collected (Additional file 4). A physician, orthoptist or optician is asking these questions before refractioning the study eye and measuring the best corrected visual acuity. The study participant then goes to the injection room. After the IVI, the participant is asked to answer the patient satisfaction questionnaire by a secretary (Additional file 5). Each study participant has an individual study booklet marked with study number and patient initials. The booklet follows the patient during the visit in the department and is otherwise kept in a locked room.

In the following visits the study participant will be asked follow-up questions regarding use of health care service (Additional file 4). The questions are asked by the nurse or physician performing the IVI.

The last visit of the 12 month period is performed similar to the baseline registration (Additional file 4). The time window for the final test is 12 ± 2 months.

The nurse or physician performing the IVI, will fill in a daily report including the number of patients and eyes treated and number of questions asked a senior consultant (Additional file 3).

Researchers will continually examine the study forms in the booklets for missing data. Missing data will be sought collected via phone calls.

### Statistical methods

The primary objective will be analyzed using a t-test for non-inferiority, comparing mean change in visual acuity from baseline to 12 months between nurses and physicians. A linear regression model for the change in visual acuity will be used to compare the treatments after adjusting for diagnosis, used as a stratification variable. Adjusting for the other stratification variable, first or follow up treatment, can be done similarly, but will depend on a sufficient number of included patients in each of the two groups. The data will be analyzed and presented according to the CONSORT guidelines for reporting noninferiority trials [17].

Costs will be examined by analyzing differences between nurses and physicians in total cost per patient, health service cost per patient and hospital cost per patient. Imputation will be used on missing data. Sensitivity analyses will be performed to assess parameter uncertainty.

### Sample size

The sample size is calculated for the noninferiority study comparing change in visual acuity for patients treated by nurses to those treated by physicians.

#### Sample size considerations

In contrast to earlier studies comparing the effect of two different anti-VEGF drugs or the effect of different anti-VEGF treatment strategies, we want to test the effect of two different professions performing the treatment. Historical data from large randomized clinical AMD trials found the mean change in visual acuity during the first year of treatment to be 6–7 letters (1,2–1,4 lines) [18]. The CATT study was as the present study designed as a noninferiority study. As the noninferiority margin (δ_L_) should be less than the observed change in visual acuity, five letters (one line on the visual acuity chart) was chosen as the acceptable difference for the tested treatment to be considered noninferior to the reference treatment. However, in a study of the safety of nurse-administered IVI, a noninferiority margin of five letters may be considered too wide.

First the study population in the present study is not as homogenous as the before mentioned study since it includes both patients with AMD, RVO and DME. There is an anticipated difference in treatment response in these three conditions. Second, the majority of study participants will already have received several IVI before inclusion in the study and in these patients we do not expect major changes in visual acuity during the study period. Third, we should consider the ethical aspect of having a wide noninferiority margin; if it is right to sacrifice visual acuity to gain the possible benefit of cost savings by nurses treating the patients. Taking these aspects into consideration, we find that the noninferiority margin should not exceed three letters. In other words, the present study will test whether IVI administered by nurses is not less effective than treatment by physicians, by more than three letters.

We assume that the standard deviation (SD) of the distribution of changes, σ, will be 10 letters. This is again less than in the studies forming the basis for IVI of anti-VEGF, finding a SD of 15 letters reasonable the first year. The second year, however, the standard deviation, dropped to 11 letters and in our study we do assume 10 letters would be reasonable [8].

The anticipated true difference between the treatment groups is 0. That is, the effect of the treatment is expected to be equal in the two groups.

#### Sample size and power calculation

The sample size is calculated by using the abovementioned assumptions and the sample size formula for comparing two means in a noninferiority trial (SPSS Sample Power 3).

Choosing a noninferiority margin δ_L_ =3, standard deviation SD = 10 and a significance level of 0.05, 140 participants is required in each group to have a power of 80 % to reject the null hypothesis, H_0_ ≤ - δ_L_ and conclude that nurses are treating the patients equally safe as or better than physicians, if the null hypothesis is true.

We anticipate the percentage of patients completing the final visit at 12 months to be 92 % as a dropout of 8 %, including patient death and illness, is not uncommon in similar trials. This means that to make sure that 140 participants complete a 12-month observation period; at least 152 patients should be included in each of the study arms.

## Discussion

The primary objective of the study is to evaluate safety of nurse-administered IVI of anti-VEGF compared with physician-administered IVI. Anti-VEGF IVI represents a considerable workload on ophthalmologic clinics and will probably continue to do so in the near future. Extending the roles of other health workers may relieve this workload from ophthalmologists and several clinics have had good experience training nurses to perform IVIs independently [11–14]. However, none have as far as we know, investigated the safety, cost and patient acceptance of independent IVI administration by trained nurses in a RCT.

We have chosen visual acuity as the primary outcome of the present study. The major goal is to investigate safety of nurse-administered IVI and visual acuity is recommended by health authorities in clinical trials when investigating this outcome [19]. Furthermore, most patients probably want to be sure that nurse-administered IVI does not pose any increased risk of deteriorating their sight.

It is conceivable that several aspects of the IVI procedure may affect visual acuity: the injection technique may be unsatisfactory performed so that the drug may not be administered correctly into the tissue where it acts, contraindications may be misinterpreted putting the patient at risk of complications or the treatment plan may be misinterpreted so that patients receive IVI with too lengthy intervals. The training and certification of the nurses is a key in this context, and the present study is in many ways a test of whether the training of nurses was adequate or not.

We believe that the design of the study, the randomization procedure and outcome measurements will be of sufficient strength and quality to evaluate if nurses are performing IVIs as safe as the standard care. Both newly remitted patients and patients treated before are invited to participate in the study and our experience so far is that it is easier including patients familiar with the treatment than the newly remitted patients. If few newly diagnosed patients are included, the interpretation of results will be for the follow-up patients only.

The present study is not dimensioned to evaluate whether there is an increased risk of complications that need treatment, since the rates of these complications are very low. Given that nurse-administered IVI is safe, we find the secondary outcomes equally relevant and of great importance to examine adverse events, patient satisfaction and economic aspects.

### Trial status

The first patient was recruited to the trial March 1. 2015 and recruitment ended December 2015. Data collection will continue until January 2017.

## Additional files

Additional file 1: EQS assistant. Instructions for the assisting nurse, preparing the patients for intravitreal injections. (PDF 202 kb) Additional file 2: EQS operator. Instructions for the operator performing the intravitreal injections. (PDF 188 kb) Additional file 3: Daily report. Recording the number of patients and eyes treated and the number of questions asked a senior consultant. (PDF 172 kb) Additional file 4: Patient report. Recording the use of health care service. (PDF 97 kb) Additional file 5: Patient satisfaction questionnaire. A short questionnaire about satisfaction with the treatment asked after the first and last visit. (PDF 98 kb)

## Abbreviations

AMD: Age-related macular degeneration
anti-VEGF: anti-vascular endothelium growth factor
DME: Diabetes macular edema
IVI: Intravitreal injection
RCT: Randomized controlled trial
RVO: Retinal vein occlusion
SD: Standard deviation
