# Supplementary material for: Nurse-administered intravitreal injections of anti-VEGF: study protocol for noninferiority randomized controlled trial of safety, cost and patient satisfaction
Source: BMC Ophthalmol. 2016 Oct 1;16:169. doi: 10.1186/s12886-016-0348-4 (PMC5045663; doi:10.1186/s12886-016-0348-4)
Supplement: Additional file 1: — EQS assistant. Instructions for the assisting nurse, preparing the patients for intravitreal injections. (PDF 202 kb) [file 12886_2016_348_MOESM1_ESM.pdf]

The Intravitreal Injection Clinic at Department of Ophthalmology,

St. Olav's Hospital, Trondheim University Hospital, Norway.

Instructions for the assistant.

Introduction:

* Intravitreal injections (IVI) of medication is a treatment method used in an ever increasing number of eye conditions.

* The largest group of patients receiving treatment with IVI containing growth factor inhibitors (anti VEGF) is patients with Age related macular degeneration (AMD), followed by patients with retinal vein occlusions, diabetes macular edema and other retinal diseases. The anti-VEGFs injected are Avastine (bevacizumab), Eylea (aflibercept) and Lucentis (ranibizumab).

* In addition to anti-VEGF, corticosteroids are injected in fluid form (Triesence) and as a depot tablet (Ozurdex).

* The treatment is implemented by authorized personnel in a facilitated treatment room at the out-patient clinic.

Objective and Scope:

* This procedure aims to ensure equality of information, observation and preparation for treatment of the patient given intraocular injections, and to minimize discomfort.

Responsibility:

*Physician/nurse at the out-patient clinic.

Working description:

Performed by:	Working task
Physician/nurse	**Preparing the room for injection**:<br><br>* Take a copy of the patient list from the drawer in room 1019.<br><br>* From the refrigerator in the eye policlinic storage room, collect Avastine 25 mg/ml and Lucentis 10mg/ml and Eylea 40 mg/ml and Triesence 40mg/ml. Take Orurdex in the cupboard of the injection room.<br>* Avastine, Lucentis and Eylea are later stored in the fridge on the 6th floor after use.<br><br>**Clothing:**<br><br>* Cover all hair with a cap. Remove watch and jewelry.<br><br>* While filling syringes and working in the operation room, the physician/nurse should use a cap and surgical mask.<br><br>* The wash basin, working trolley, assistance table and the upper parts of the treatment chair is disinfected with alcohol disinfectant (70% Antibac).<br><br>* Cover the assistance table with an adhesive edged sterile drape.<br><br>* Place sterile syringes and syringe needles on to the sterile drape.<br><br>* Wipe the vial with alcohol disinfectant (Alkotip) before extracting the medicine.<br><br>* Prepare the syringes in accordance to the sterile procedure.<br><br>**The extraction /portioning of the medication:**<br><br>* A codan spike is inserted in to a bottle of Avastine 25mg/ml.<br>* Extract up to 0.15ml of Avastine in a syringe Omnifix-F.<br>* Cannula BD Mikrolance 3.30G, 0.3x13mm for injection.<br><br>* For the Extraction and portioning of Lucentis 10mg/ml (Individual set in each package).<br>* 1 syringe 10mg/ml is divided into 2 insulin syringes (micro-Fine, 0.3mm x 8mm.<br>* Cannula BD Microlance 3.30G, 0.3 x 13mm for injection.

	For the extraction/ portioning of Eylea 40mg/ml (Individual set in each package).<br> 1 syringe Eylea 40mg/ml is divided into 3 insulin syringes MikroFine 0.3mm x 8 mm.<br>* Cannula BD Microlance 3.30 G, 0.3 x 13mm for injection.<br><br>* Other medication is prepared immediately before injection.
Physician/nurse	**Storage:**<br>* The prepared syringes with the medication are aligned on the sterile drape with some distance between them.<br><br>* A Sterile drape is place over them for protection.<br><br>* Medication used after lunch is put into a sterile bag and placed in a refrigerator on the 6th floor.
Nurse/Doctor	**Patient treatment:**<br><br>* The patient is called in from the waiting room on the 6th floor.<br><br>* Check the patient`s personalia and identify the eye to be injected. Ask if he has received injections before.<br><br>* Check the patient`s eye for infection (conjunctivitis/blepharitis). If you are in doubt; inform the operator.<br>Patients with infections are informed of the treatment, given relevant medication and the brochure: Treatment of eye lock infection. ; The IVI is postponed until the treatment is finished.<br><br>* Remove any make-up with make-up remover.<br><br>* Put the cap on head and place the gauze by the correct eye.<br><br>*Drip the relevant eye with Oxibuprokain eye drops, then 1 drop of Betadine 5%. (NB! Iodine allergy)<br><br>* Before the patient is escorted into the injection room, ask the patient discreetly about his/her recent health status:<br>*With recent gastric flu/ diarrhea; the patient must be symptom free the last 2 days.<br>* With upper respiratory infection, coughing and runny nose; postpone the injection until the patient is symptom free.<br>* With infections that require antibiotics; the IVI is postponed until the a.b. treatment is finished.<br>* With recent heart attack or stroke; the IVI is postponed for a month.

	* Safe surgery check list.<br><br>* Position the patient on the operating chair.<br><br>*Give the patient the 2nd & 3rd drops of Oxibuprokain.<br><br>* The eye and eyelashes are then soaked with Betadine 5%, followed by wiping the upper and lower eyelids well, using Betadine soaked gauze balls.<br><br>* After the injection, apply Betadine 5% before removing the eye speculum.<br><br>* Viscotears can be given to ease discomfort after the injection.<br><br>NB: Iodine allergy<br><br>* Chloramphenicol eye drops are administered instead of Betadine. The eye is cleansed with sterile gauze balls/or Sugi soaked with chlorhexidinalcohol 5mg/ml.<br><br>* Drip with chloramphenicol eye drops after the injection before removing the eye speculum.

At the end of the day

* Medicine bottles are marked with the days date and stored for 4 weeks in the disinfection room.

* Remember to re- charge the battery of the operation chair.

* The treatment room is made tidy and equipment replaced.

* Used equipment is transported to a collection point to be collected by porters, at lunch and by the end of the day.

Related documents

Info to those who have received eye medication.

Info about treatment with anti-VEGF.

Treatment of eyelid infection. Good eyelid hygiene.
