# Supplementary material for: Nurse-administered intravitreal injections of anti-VEGF: study protocol for noninferiority randomized controlled trial of safety, cost and patient satisfaction
Source: BMC Ophthalmol. 2016 Oct 1;16:169. doi: 10.1186/s12886-016-0348-4 (PMC5045663; doi:10.1186/s12886-016-0348-4)
Supplement: Additional file 2: — EQS operator. Instructions for the operator performing the intravitreal injections. (PDF 188 kb) [file 12886_2016_348_MOESM2_ESM.pdf]

The Intravitreal Injection Clinic at Department of Ophthalmology,

St. Olav's Hospital, Trondheim University Hospital, Norway.

Instructions for the operator.

Introduction:

* Intravitreal injection (IVI) of medication is a treatment method used in an ever increasing number of eye conditions.

* The largest group of patients receiving treatment with IVI containing growth factor inhibitors (anti VEGF) is patients with Age related macular degeneration (AMD), followed by patients with retinal vein occlusions, diabetes macular edema and other retinal diseases. The anti-VEGFs injected are Avastine (bevacizumab), Eylea (aflibercept) and Lucentis (ranibizumab).

* In addition to anti-VEGF, corticosteroids are injected in fluid form (Triesence) and as a depot tablet (Ozurdex).

* The treatment is implemented by authorized personnel in a facilitated treatment room at the out-patient clinic.

Intention and scope:

* The procedure aims to ensure equal treatment of patients receiving IVI in relation to information, the sterile procedure and for correct injection technique.

Responsibility:

* Physician /nurse authorized to execute IVI implementation.

Working description:

Performed by	Working task
Physician/nurse who is authorized to give injections	**Before starting the treatment**<br><br>* Rings, watches and jewelry are removed.<br>* Put on a cap and a surgical mask.<br>* Start with a surgical hand wash (in the morning- thereafter antimicrobial disinfectant) and sterile gloves. * Collect data about the patient from the electronic patient records (EPR). Ensure that the right patient receives the right treatment in the correct eye.<br><br>**Implementation**<br><br>* The operator confirms the information given by the assistant in accordance with the safe surgery check list.<br><br>* The sterile intravitreal instrument basket containing the eye speculum and caliper is prepared.<br><br>* Check that the syringe is filled with the right amount of medicine and that air bubbles are removed.<br><br>* Insert the eye speculum.<br><br>* Make a mark with the caliper 3.5mm from the limbus.<br><br>* Place the needle point in the cavity perpendicular to the bulbous.<br><br>* Insert the needle into the eye and inject the medicine.<br><br>* Remove the eye speculum after the assistant has applied Betadine 5% eye drops.<br><br>**Further course of treatment**<br><br>* Ensure the patient is informed of the continuing treatment.<br>* Fill in the form, planning for the next visit.<br>* Report in the EPR.

Patient information

* Ask the patient if he has questions concerning the treatment.

* Ensure that the patient has understood the implications of good hygiene principles for the first 3 days.

* The patient is informed to make contact if symptoms of redness or pain occur.

* Ensure the patient has been given the brochure: Information for those who have received eye medication.

Related documents

Info for those who have received eye medication.

Info about treatment with anti-VEGF

Treatment of eyelid infection. Good eyelid hygiene.
