# Supplementary material for: Nurse-administered intravitreal injections of anti-VEGF: study protocol for noninferiority randomized controlled trial of safety, cost and patient satisfaction
Source: BMC Ophthalmol. 2016 Oct 1;16:169. doi: 10.1186/s12886-016-0348-4 (PMC5045663; doi:10.1186/s12886-016-0348-4)
Supplement: Additional file 3: — Daily report. Recording the number of patients and eyes treated and the number of questions asked a senior consultant. (PDF 172 kb) [file 12886_2016_348_MOESM3_ESM.pdf]

**Daily report** **Week no:**	Monday	Tuesday	Wednesday	Thursday
**Today's date, DD.MM.YY**				
**Doctor or nurse administers IVI? 1=doctor, 2=nurse**				
**Scheduled no. of IVI / eyes**				
**No. of extra unscheduled IVI / eyes during the day**				
**Total no. of IVI / eyes injected today**				
**No. of patients given an IVI today**				
**No. of patients that did not show up to their scheduled appointment**				
**No. of patients not given an IVI because of blepharitis**				
**Total no. of questions asked to an ophthalmologist today**				
**No. of questions asked regarding infection**				
**No. of questions asked regarding future follow-up**				
**No of questions regarding other topics (not specified above)**				
**Specify questions asked regarding other topics**				
