# Supplementary material for: Nurse-administered intravitreal injections of anti-VEGF: study protocol for noninferiority randomized controlled trial of safety, cost and patient satisfaction
Source: BMC Ophthalmol. 2016 Oct 1;16:169. doi: 10.1186/s12886-016-0348-4 (PMC5045663; doi:10.1186/s12886-016-0348-4)
Supplement: Additional file 4: — Patient report. Recording the use of health care service. (PDF 97 kb) [file 12886_2016_348_MOESM4_ESM.pdf]

**Patient report** Fill in the white fields only	Baseline/ IVI no. 1	IVI no. 2	IVI no. 3	IVI no. 4	IVI no. 5	IVI no. 6	IVI no. 7	IVI no. 8
Date of injection, DD.MM.YY								
Does the patient come alone or with a companion?<br>0 = alone, 1 = with a companion								
Has the patient seen his general practitioner since the last IVI? Mark no. of visits.								
Has the patient seen his ophthalmologist since the last IVI? Mark no. of visits.								
Patient is living:<br>1 = in his own home<br>2 = with relatives<br>3 = in a nursing home<br>4 = in a municipal shelter<br>5 = other								
Is the patient receiving any nursing home care from the municipality? Note no. of hours per week (rounded up to the nearest hour).								
Is the patient receiving any home services from the municipality? Note no. of hours per week (rounded up to the nearest hour)								

**What is the patients source of subsistence?**<br>**1 = Retirement pension**<br>**2 = Gainful employment**<br>**3 = Employment scheme**<br>**4 = Unemployed**<br>**5 = Permanent disability benefit**<br>**6 = Other**								

Last IVI in study
