# Supplementary material for: Nurse-administered intravitreal injections of anti-VEGF: study protocol for noninferiority randomized controlled trial of safety, cost and patient satisfaction
Source: BMC Ophthalmol. 2016 Oct 1;16:169. doi: 10.1186/s12886-016-0348-4 (PMC5045663; doi:10.1186/s12886-016-0348-4)
Supplement: Additional file 5: — Patient satisfaction questionnaire. A short questionnaire about satisfaction with the treatment asked after the first and last visit. (PDF 98 kb) [file 12886_2016_348_MOESM5_ESM.pdf]

When thinking about the time you have spent with us today, the preparation for the operation, the injection and the information given; what is your general impression? How satisfied are you with today’s visit (mark your choice):<br><br>1. Not satisfied at all<br>2. Not very satisfied<br>3. Satisfied to some extent<br>4. Satisfied<br>5. Very satisfied<br><br>What can we do to make you more satisfied?
How confident did you feel during the treatment today? (mark your choice)<br><br>1. Not safe at all<br>2. Not very safe<br>3. Safe to some extent<br>4. Safe<br>5. Very safe<br><br>What can we do to make you feel more safe?
QUESTION ASKED AFTER THE LAST STUDY VISIT:<br><br>Who do you think have given you injections during the past year?<br><br>1. physicians<br>2. nurses<br>3. I am not sure
